# Microbial Community Response to Granular Peroxide-Based Algaecide Treatment of a Cyanobacterial Harmful Algal Bloom in Lake Okeechobee, Florida (USA)

**DOI:** 10.3390/toxins16050206

**Published:** 2024-04-26

**Authors:** Forrest W. Lefler, Maximiliano Barbosa, David E. Berthold, Rory Roten, West M. Bishop, H. Dail Laughinghouse

**Affiliations:** 1 Fort Lauderdale Research and Education Center, University of Florida—IFAS, Davie, FL 33314, USA; flefler@ufl.edu (F.W.L.); mbarbosa@ufl.edu (M.B.); dberthold@ufl.edu (D.E.B.); 2 SePRO Research and Technology Campus, 16013 Watson Seed Farm Road, Whitakers, NC 27891, USA; roryr@sepro.com (R.R.); westb@sepro.com (W.M.B.)

**Keywords:** *Microcystis*, cyanobacteria, bacteria, protist, 16S rRNA, HAB management, microcystin

## Abstract

Cyanobacterial harmful algal blooms (cyanoHABs) occur in fresh water globally. These can degrade water quality and produce toxins, resulting in ecological and economic damages. Thus, short-term management methods (i.e., algaecides) are necessary to rapidly mitigate the negative impacts of cyanoHABs. In this study, we assess the efficacy of a hydrogen peroxide-based algaecide (PAK^®^ 27) on a *Microcystis* dominated bloom which occurred within the Pahokee Marina on Lake Okeechobee, Florida, USA. We observed a significant reduction in chlorophyll *a* (96.81%), phycocyanin (93.17%), and *Microcystis* cell counts (99.92%), and a substantial reduction in microcystins (86.7%) 48 h after treatment (HAT). Additionally, there was a significant shift in bacterial community structure 48 HAT, which coincided with an increase in the relative abundance of photosynthetic protists. These results indicate that hydrogen peroxide-based algaecides are an effective treatment method for cyanoHAB control and highlight their effects on non-target microorganisms (i.e., bacteria and protists).

## 1. Introduction

Cyanobacterial harmful algal blooms (cyanoHABs) occur in eutrophic waters and can degrade water quality, potentially leading to ecological and economic damages [1]. These blooms can produce a myriad of cyanotoxins and taste and odor compounds [2] which have detrimental effects on wildlife, livestock, and human life via direct consumption or the aerosolization of toxins [3,4]. CyanoHABs are often dominated by the genus *Microcystis,* but can also be dominated by other taxa, and have been increasing in many areas over the last few decades due to eutrophication and climate change [5,6,7,8,9,10,11]. Species of *Microcystis*, especially *M. aeruginosa* (Kützing) Kützing, have caused large cyanoHABs in freshwater lakes around the globe, including large lakes such as Lake Erie, Lake Taihu, and Lake Okeechobee [7]. These cyanoHABs warrant fast and effective control methods.

Nutrient (i.e., nitrogen and phosphorous) input reduction is often an effective and desired method to control and mitigate cyanoHABs; however, this can take many years to achieve due to diffused sources [12] and may not result in a decrease in cyanoHABs in a water body because of legacy accumulation [13]. Thus, short-term strategies, such as algaecide applications, are necessary to manage cyanoHAB events as they can quickly and efficiently restore critical uses of the water resource [14,15,16,17,18]. Several formulations of algaecides exist, with copper- and peroxide-based algaecides being the most common for the treatment of cyanobacteria [19]. Peroxide-based algaecides can also have different forms of the active ingredient; solid formulations use sodium carbonate peroxyhydrate as their active ingredient while liquid formulations use a combination of hydrogen peroxide and peroxyacetic acid. Peroxide-based algaecides have shown negligible toxicity towards a plethora of non-target organisms when applied at label rates [20,21,22] and, as opposed to copper, decay leaving no residues in the treated system [14,23]. The application of peroxide-based algaecides may result in an increase in aqueous toxins (i.e., dissolved, extracellular), but an overall reduction in total (i.e., extracellular and intracellular) cyanotoxin concentrations shortly after treatment, either due to oxidation, dilution, adherence to proteins, or bacterial degradation [14,15,24,25]. Furthermore, cyanobacteria have proved to be more susceptible to peroxide-based algaecides than other eukaryotic phytoplankton groups, which may shift toward a beneficial algal assemblage post-application [14,25,26].

Much work has been performed on assessing the efficacy of peroxide-based algaecides on site-collected cyanobacteria from field blooms [15,18], in situ mesocosms [16,17], and even small lakes [14,21,27,28]. However, most studies primarily focus on the effects on cyanobacterial abundance, toxin degradation, or non-target organisms and occur under confined laboratory conditions or mesocosms. Recent works have begun to assess the effects of peroxide treatments on the bacterioplankton community in small lakes and have shown that treatments only temporarily affect the overall bacterial community structure [28]. In this study, we assessed the effects of a peroxide-based algaecide treatment in situ on a *Microcystis*-dominated cyanoHAB within a marina in Lake Okeechobee, including the effects on non-target bacteria and microbial eukaryotes (protists) via 16S and 18S rRNA metabarcoding, phytoplankton counts, and toxin analysis. This study represents an operational treatment of a cyanoHAB in a real-world scenario and provides critical information regarding ecosystem alterations following operational management.

## 2. Results and Discussion

### 2.1. Effects of Peroxide-Based Algaecide on Cyanobacterial Abundance and Toxins

**Figure 1:**
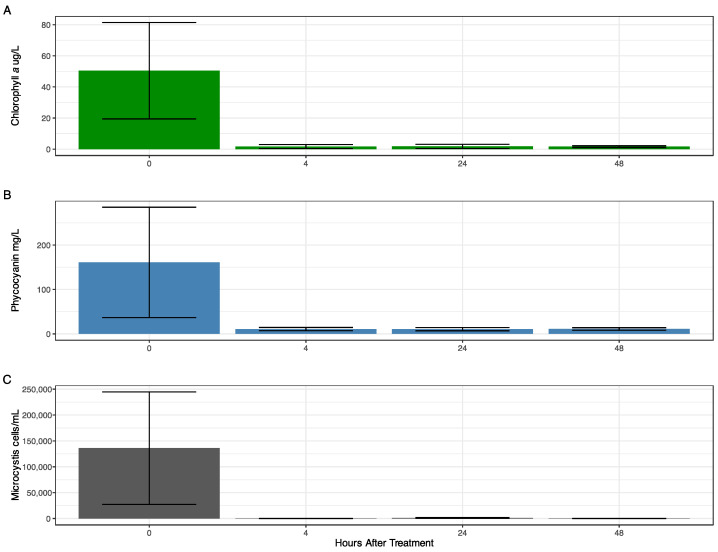
Bar plots indicating the average measure of cyanobacterial abundance through time. Error bars indicate standard error. (**A**): Chlorophyll *a* concentration. (**B**): Phycocyanin concentration. (**C**): *Microcystis* counts.

Before the treatments, *Microcystis aeruginosa* was the dominant taxon forming the bloom. *Microcystis* cell abundance (as counts), chlorophyll *a*, and phycocyanin varied within the marina before treatment, with the highest abundance at Site 4 and lowest at Site 2 for all metrics (Figure S1). Four hours after treatment (HAT), cell abundance measures decreased throughout all sites in the marina and remained at these lower levels even at 48 HAT (Figure S1). When averaging the cell abundance and chlorophyll *a* and phycocyanin concentrations from all sites in the marina, there was a substantial decrease in phycocyanin levels (93.17%) and a significant decrease (*p* ≤ 0.05) in cell abundance (99.92%) and chlorophyll *a* concentration (96.81%) 4 HAT (Figure 1). A rapid decline in cyanobacterial abundance due to peroxide treatment has been documented in several works [14,27,28], with variable effects on the different bloom-forming cyanobacterial genera. However, different bloom forming taxa have disparate sensitivities to peroxides [27]. *Dolichospermum* has been shown to be more susceptible to peroxides than *Planktothrix*, likely due to the ability of *Planktothrix* to produce peroxidases [28]. *Microcystis* has been shown to be very susceptible to peroxide treatments both in this work and in others (e.g., [27]); although cultured isolates, especially single-celled cultures, are likely more susceptible to treatment than colonies. Several works [29,30,31] have shown that *Microcystis* genomes, as both cultured isolates and meta-genome assembled genomes, lack the capabilities to produce many, but not all, peroxidases and catalases. This decreases their ability to resist increased peroxide concentrations; especially in toxin producing strains [31]. Furthermore, Smith et al. [29] observed the transcriptomic response during a *Microcystis*-dominated bloom in Lake Erie and found there to be little to no expression of known peroxidases and catalases from *Microcystis*, rather, these were produced by the associated bacteria.

**Figure 2:**
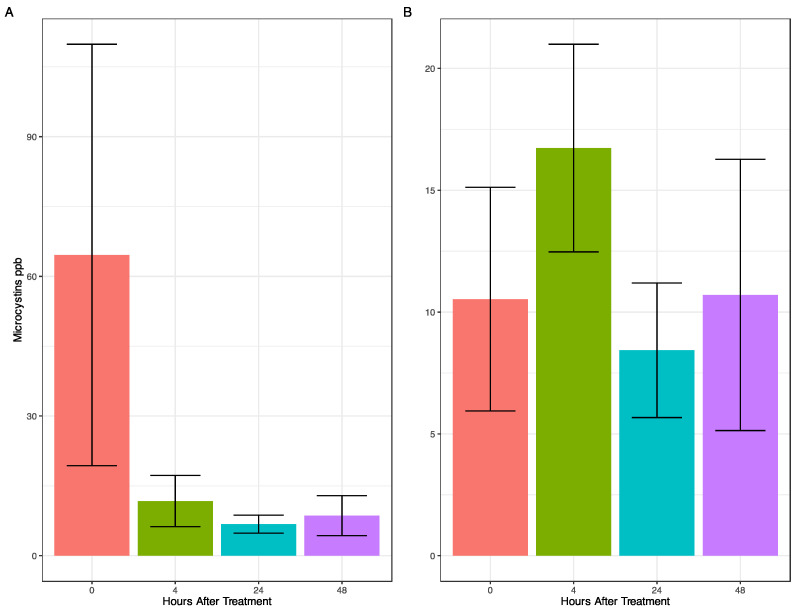
Bar plots indicating the mean microcystin concentration through time. Colors indicate hours after treatment (HAT). Error bars indicate standard error. (**A**) Mean total microcystins. (**B**) Mean aqueous microcystins.

Prior to treatment, total microcystin concentrations varied from 197 ppb at Site 4 to 1.68 ppb at Site 2 (Figure S2A) and aqueous microcystins varied from 20.9 ppb at Site 3 to 2 ppb at Site 2 (Supplementary Figure S2B). Total microcystins were reduced in the marina 4 HAT and remained low (Figure 2A), although there was a rise in aqueous microcystins, which returned to starting concentrations 48 HAT (Figure 2B). Aqueous microcystins decreased below initial levels 48 HAT at Sites 1 and 2, with no change at Site 3, and an increase at Site 4 where the bloom was most dense (Figure S2B). Peroxide-based algaecides, such as PAK^®^ 27, are known to oxidize microcystins [14,23,32,33,34], resulting in the rapid decrease in total microcystin concentration, although the decrease could be due to other factors (e.g., bacterial degradation as the peroxide concentration may not have been high enough for this effect [35]. The increase in aqueous microcystins 4 HAT (Figure 2B) corresponds to the significant decrease in *Microcystis* cell abundance (Figure 1C). Cell-bound toxins are known to be released into the water column following algaecidal treatment, temporarily increasing the aqueous concentration [25,36]. In contrast to total microcystins, which rapidly decreased in concentration (86.7%) and remained low, aqueous concentrations of microcystins remained roughly the same concentration within the marina throughout the course of the treatment, showing a similar trend to that of Barrington et al. [25].

### 2.2. Community Composition

**Figure 3:**
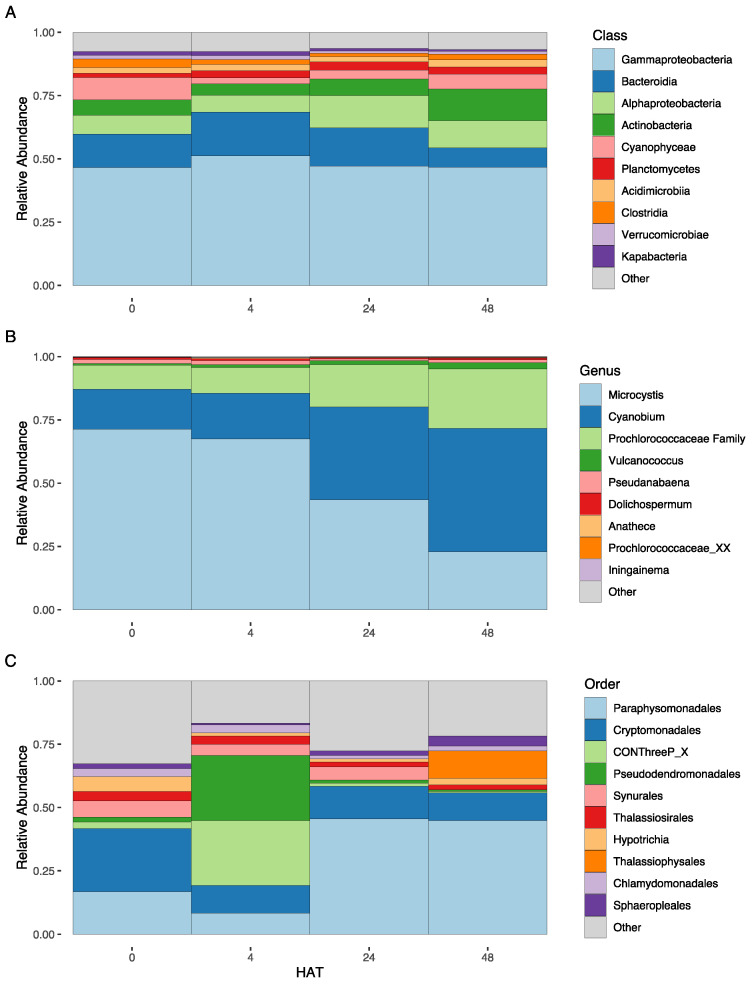
Bar plots showing the relative abundance of the (**A**): bacterial classes; (**B**): cyanobacterial genera; and (**C**): protistan orders, found during the study.

For the bacterial 16S rRNA primers, a total of 1,319,296 high-quality reads were generated after low-quality reads were filtered. A total of 5492 unique ASVs were recovered after removal of non-target taxa and 209 of the total ASVs were cyanobacterial. Overall, ASV’s corresponding to cyanobacteria made up a small percentage of the reads and most of the reads belonged to bacterial classes such as Gammaproteobacteria, Bacteroidia, and Alphaproteobacteria (Figure 3A). There was an increase in Alphaproteobacteria and Actinobacteria and a decrease in Bacteroidia 24 HAT, potentially indicating that hydrogen peroxide negatively effects Bacteroidia. There were many bacterial families that increased (e.g., Clade−III, Comamonadaceae, Sphingomonadaceae, Sporichthyaceae, and Weeksellaceae) and decreased (e.g., Chromobacteriaceae, Flavobacteriaceae, and Rhodocyclaceae) in relative abundance after treatment (Figure S3). The cyanobacteria, class Cyanophyceae, decreased 4 and 24 HAT, but began to recover 48 HAT (Figure 3A). However, there was a decrease in *Microcystis* relative abundance, with picocyanobacteria belonging to the Prochlorococcaceae, primarily as *Cyanobium*, dominating 48 HAT (Figure 3B). This is in line with the phytoplankton counts, which show the decrease in *Microcystis* (Figure 1C). Those bacterial groups which increased after treatment may have increased due to their ability to produce peroxidases, providing them protection against peroxide treatment. The bacterial groups which decreased may lack the ability to produce peroxidases, and thus declined in response to treatment, although they may have been outcompeted by those bacterial groups which were able to take advantage of the organic matter released from decaying cyanobacteria [37]. Previous work by Lusty and Gobler [38] has shown that total bacterial abundances also decrease due to peroxide treatments. However, the changes in community structure observed in this study were less intense compared to previous works [17,38] and may be explained by differences in systems (i.e., open water in a large lake vs. ponds and mesocosms). Further investigation is required to determine if the changes in the bacterial community structure were due to the death of the cyanobacteria and related release, oxidative stress from hydrogen peroxide, or post-treatment effects (e.g., increased water clarity).

For the protistan 18S rRNA primers, a total of 136,329 high-quality reads were generated after low-quality reads were filtered. A total of 664 ASVs were recovered after removal of non-protistan taxa. Taxonomic assignment yielded ASVs affiliated with 20 phyla belonging to seven supergroups (Stramenopiles, Alveolata, Hacrobia, Archaeplastida, Rhizaria, Opisthokonta, and Amoebozoa). Protist communities were dominated by an unclassified Paraphysomonadales, Cryptomonadales, CONThreeP_X, Pseudodendromonadales, Synurales, and Thalassiosirales, representing over 50% of the relative sequence abundance at all time points (Figure 3C). There was a noticeable shift at 4 HAT, with an increased abundance of CONThreeP_X and Pseudodendromonadales (Figure 3C). However, at 24 and 48 HAT these orders had decreased and the community became dominated by the group Paraphysomonadales. Chen et al. [39] found that the use of hydrogen peroxide to treat cyanobacterial blooms resulted in an increase in photosynthetic eukaryotes such as Chlorophyta, Bacillariophyta, and Euglenophyta. Our study demonstrates similar results. Although there was an initial drastic change in the composition of the protist communities after peroxide treatment in our study, with the heterotrophic groups CONThreeP and Bicoecea dominating two of the locations 24 HAT, at 48 HAT, three of the four locations became dominated by an unclassified Ochrophyte and were composed of similar groups of protists, while the last site was dominated by the photosynthetic Bacillariophyta and Chlorodendrophyceae. The unclassified Ochrophyte can potentially be a photosynthetic organism, such as Bacillariophyta, but further analyses are required. Our results support the hypothesis that hydrogen peroxide treatment can lead to the dominance of photosynthetic protists in microbial eukaryotic communities.

**Figure 4:**
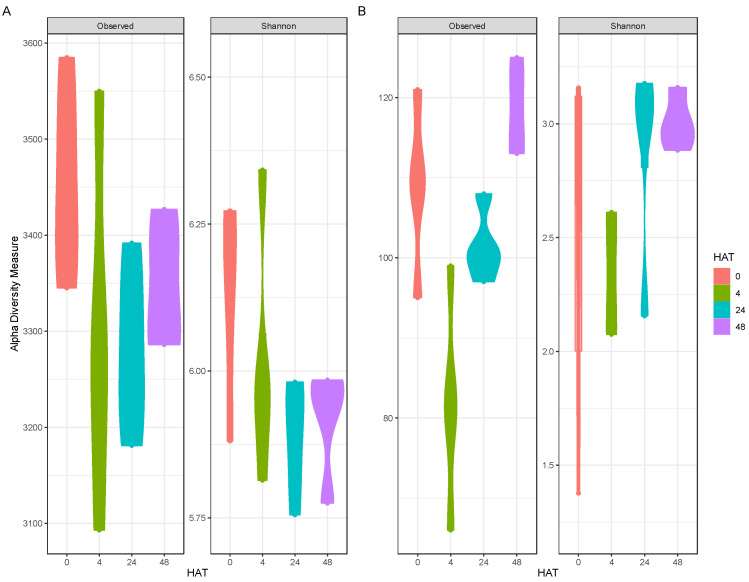
Alpha diversity of (**A**): bacterial and (**B**): cyanobacterial communities after treatment. Colors indicate season, dots indicate sample, bar represents median.

Alpha diversity, based on both diversity indices, decreased 4 HAT for both bacterial and cyanobacterial communities (Figure 4). For the bacterial communities, alpha diversity decreased, non-significantly, 4 and 24 HAT, based on both alpha diversity metrics (Figure 4A) and began to increase at 48 HAT. For the cyanobacterial communities, alpha diversity significantly decreased 4 HAT for observed diversity, and non-significantly for Shannon (Figure 4B). Alpha diversity began to increase 24 HAT based on both alpha diversity metrics (Figure 4B) and exceeded the initial diversity at 48 HAT. In contrast to results from Piel et al. [28], there were no significant differences in alpha diversity in the bacterial alpha diversity. However, the cyanobacterial communities from Piel et al. [28] were more diverse than this *Microcystis* dominated bloom, and certain taxa were seen to be more susceptible to peroxide treatment.

**Figure 5:**
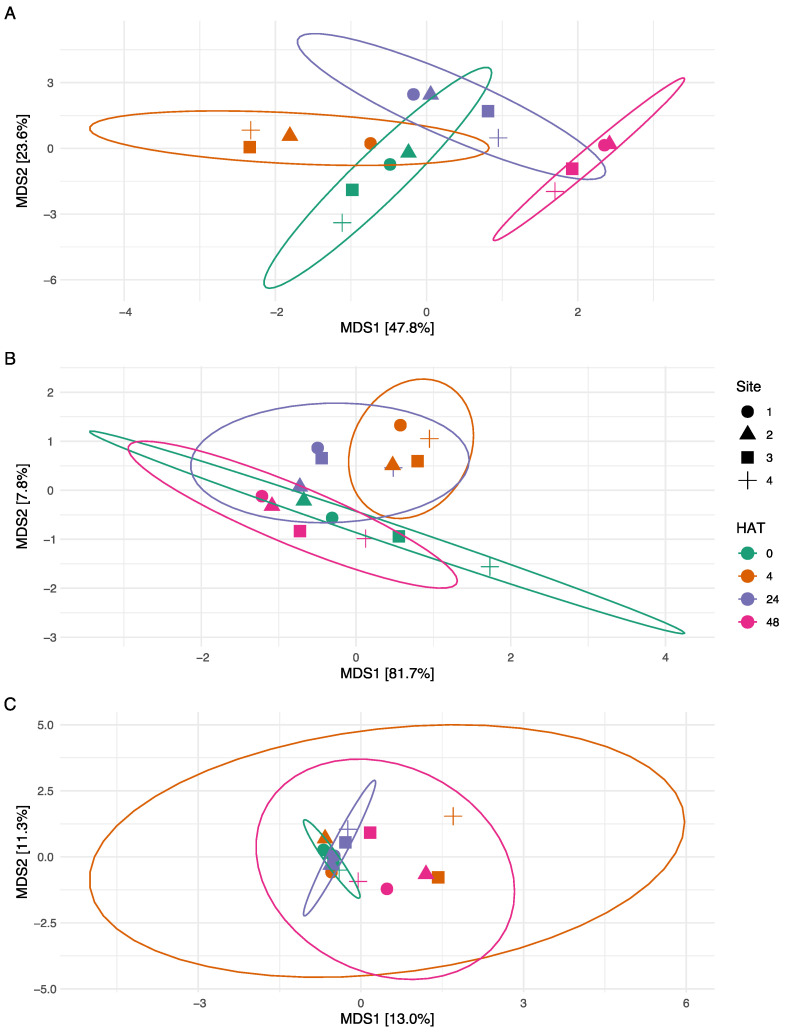
Non-metric Multidimensional Scaling ordination, within two dimensions of the (**A**): bacterial communities; (**B**): cyanobacterial communities; (**C**): protistan communities. Points indicate individual samples; colors indicate hours after treatment.

For the bacterial community, there was an overlap between the community at 0, 4, and 24 HAT, with the 48 HAT communities clustering separately (Figure 5A). An ANOSIM revealed that all communities were significantly different from one another (*p* < 0.05). The cyanobacterial communities did not show the same clear clustering as the bacterial communities (Figure 5B), and the communities at 4, 24, and 48 HAT did not significantly differ from the initial community. The PCoA analysis showed a considerable overlap between protist communities based on HAT (Figure 5C). However, ANOSIM results indicated that there was a significant difference among protistan communities based on HAT (*p* < 0.01). Specifically, HAT 0 was significantly different to HAT 24 and HAT 48, and HAT 4 was significantly different to HAT 48 (*p* < 0.05).

## 3. Conclusions

These results confirm that granular peroxides are a viable, fast-acting treatment option to manage *Microcystis*-dominated cyanoHABs. The treatment led to a significant reduction in algal biomass, as well as *Microcystis* cell abundance, chlorophyll *a*, and phycocyanin, and a substantial reduction in total microcystins. Despite this reduction in *Microcystis* and a shift to pico-cyanobacterial dominance, no significant shifts in cyanobacterial community structure were seen and *Microcystis* remained even 48 h after treatment. However, this bloom was dominated by *Microcystis* and picocyanobacteria and treatment of a more diverse bloom could yield different results, similar to Piel et al. [28]. Treatment of this cyanoHAB resulted in a significant shift in the bacterial community structure, although it remains unknown if this shift is due to a die-off of bacteria which are sensitive to peroxides, or an increase in bacteria which capitalized on the organic carbon released from the cyanobacterial die-off, or other related water quality alterations associated with treatments. In addition, these results demonstrate that peroxide treatments can affect protist community structure, resulting in the increased success of photosynthetic protists after treatment. Future efforts should include metagenomic or metatranscriptomic analyses of these communities to assess how peroxide-based treatments affect various groups, and how certain bacterial and eukaryotic taxa are able to proliferate after treatment.

## 4. Materials and Methods

### 4.1. Study Site, Peroxide Treatment, Sampling Information

**Figure 6:**
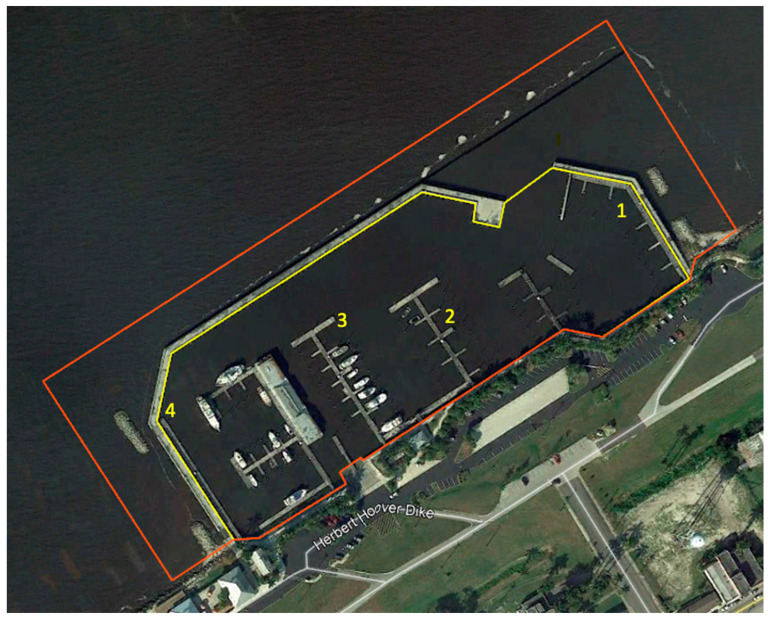
Map of sampling sites in Pahokee Marina, Pahokee, FL, USA.

During summer 2021, a cyanoHAB dominated by *Microcystis aeruginosa* occurred in the Pahokee Marina in Lake Okeechobee (Pahokee, FL, USA, 26.825767, −80.667370). The treatment area included 8.66 acres comprising the entire marina as well as an external buffer zone. Treatment was administered using a team of certified airboat pilots with side-mounted mechanical spreaders to uniformly distribute the granular peroxide (sodium carbonate peroxyhydrate) algaecide, PAK^®^ 27. The peroxide rate was calculated on the day of treatment based on the mean depth, which changes seasonally; ultimately, PAK^®^ 27 was applied at half the maximum label rate (50 lbs. per Acre Foot), which is equivalent to a concentration of 18.39 g/m^3^ (grams per cubic meter). Previous work has shown this to equate to 5 mg H_2_O_2_/L [16] (Figure 6). Subsurface water samples from four locations within the marina were collected before treatment and (0), 4, 24, and 48 h after treatment (HAT) by integrating over the top 0.3 m of the water column. Three samples (~1 L each) were taken at each designated location and homogenized for the analyses. Samples for eDNA were collected in sterile 1 L Nalgene bottles, samples of microcystins were collected in 250 mL HDPE amber bottles, and samples of pigments (chlorophyll *a* and phycocyanin) were collected in 1 L Nalgene bottles. Water samples for phytoplankton counts were fixed in acetic Lugol’s solution of 0.5% (*v*/*v*). All samples were stored on ice and transported to the laboratory and processed same day.

### 4.2. Microcystin Analysis

Samples of microcystins were evaluated as total and aqueous toxins using enzyme-linked immunosorbent assay (ELISA), following the manufacturer’s protocol (Eurofins Abraxis, Warminster, PA, USA). Samples of aqueous toxins were filtered through a 0.45 μm glass filter and frozen and samples of total toxins were frozen. Both aqueous and total samples were subjected to three freeze–thaw cycles, and total toxin samples were filtered through a 0.45 μm glass filter prior to analysis.

### 4.3. Pigment Analysis

Water samples were mixed, and a known volume was filtered onto 0.45 μm 47 mm glass fiber filters (MilliporeSigma, Burlington, MA, USA). Prior to pigment extraction, filters were subject to three freeze–thaw cycles, and protected from light exposure. Chlorophyll *a* was extracted and quantified using an ethanol extraction [40] while phycocyanin was extracted and quantified using sodium phosphate buffer, following the work presented in [41]. After extraction, pigments were quantified via spectrophotometer at their respective wave lengths.

### 4.4. Phytoplankton Counts

Lugol’s fixed samples were homogenized and a subsample was poured into Utermöhl chambers and sedimented overnight before counting [42]. Gentle sonication (25 kHz 200 Watt) was used for 1–2 min to disperse into smaller colonies or single cells when needed [43]. Phytoplankton counts were carried out using the Utermöhl method in 20 mL chambers [44] counting at least 20 random fields of view (using both vertical and longitudinal transects), with an inverted phase-contrast microscope (Olympus CKX41, Hamburg, Germany). Cell concentrations were calculated following the work presented in [42,45].

### 4.5. DNA Extraction, 16S rRNA and 18S rRNA Amplification and Sequencing

Water samples were collected and stored on ice before same-day processing in the laboratory (Fort Lauderdale Research and Education Center, University of Florida/IFAS). Samples of environmental DNA were filtered on 0.22 µm mixed cellulose filters (MilliporeSigma, Burlington, MA, USA) and DNA was extracted using protocols established by [46]. The V4–V5 hypervariable regions of the 16S rRNA were amplified using the 515FY-926R primer pair described in [47], and the eukaryotic V4 hypervariable region of the 18S rRNA was amplified using the primers EK-565F-NGS and UNonMet [48]. The 16S rRNA amplicon libraries were prepared and sequenced using paired-end (2 × 250 bp) Illumina NovaSeq (Novogene, Beijing, China). For the 18S rRNA gene, triplicate PCR reaction products per sample were pooled before purification. Pooled amplicons were purified using AMPure XP beads (Beckman Coulter Life Sciences, Brea, CA, USA) following the manufacturer’s protocols. Amplicon libraries were indexed using Nextera V2 indexing kit (Illumina, San Diego, CA, USA) following the manufacturer’s protocols. Indexed amplicon products were purified using AMPure XP beads (Beckman Coulter Life Sciences, Brea, CA, USA) following the manufacturer’s protocols. The 18S rRNA amplicon libraries were sequenced using paired-end (2 × 300 bp) Illumina MiSeq (Interdisciplinary Center for Biotechnology Research [ICBR], RRID:SCR_019152, University of Florida, Gainesville, FL, USA).

### 4.6. Sequencing Analysis

The 16S and 18S rRNA amplicon sequences were demultiplexed and assigned to specific sample IDs based on their MIDs at Novogene and ICBR, respectively, using in-house bioinformatic pipelines. DADA2 [49] was used to process raw sequences in R v4.0.0 [50]. Paired-end reads were filtered, trimmed, and merged prior to dereplication and then analyzed for the detection and removal of potential chimeras using DADA2. Non-chimeric sequences were pooled together to define amplicon sequence variants (ASVs). The 16S rRNA and 18S rRNA amplicon sequences were processed separately.

The taxonomic assignment of ASVs was based on the naïve Bayesian classifying method [51] and the 16S rRNA database CyanoSeq V1.2 [52] with SILVA 138.1 [53] was used as the bacterial reference, and for protists, the 18S rRNA database PR2 v4.13 [54] was used as reference. For the analyses of 16S rRNA samples, archaeal, chloroplast, eukaryotic, and mitochondrial ASVs were removed, and for the 18S rRNA samples, metazoans, fungi, and embryophyte ASVs were removed.

ASVs were rarified to an even depth [55] using the phyloseq package [56]; ASVs which occurred <100 times across all samples were filtered before downstream processing, except for alpha diversity analyses. The vegan package [57] was used for statistical analyses, the calculation of diversity indices, and the generation of ordinations in combination with ggplot [58]. Alpha diversity was calculated using the observed diversity and Shannon index; a Wilcoxon test was used to compare indices between each time point. No alpha diversity analyses were conducted for the protists, as the read abundance was low. Changes in the bacterial community structure were explored using Principal Coordinates Analysis (PCoA) with Aitchison distances. The protistan community structure was explored using Principal Coordinates Analysis (PCoA) with binary Jaccard distances. Using the “anosim” function of the vegan package, the analysis of similarity (ANOSIM) was conducted to assess the changes in community composition through time using 9999 permutations.

## Data Availability

Sequences were deposited in the Sequence Read Archive of the National Center for Biotechnology Information (NCBI) and made publicly available under accession number PRJNA1088079. The R code used for data analysis, including a full list of R packages, is on GitHub at https://github.com/flefler/Pahokee_MicrocystisBloom_16S_18S.

## Supplementary Materials

The following supporting information can be downloaded at: https://www.mdpi.com/article/10.3390/toxins16050206/s1, Figure S1: Bar plots indicating measures of cyanobacterial abundance grouped by location through time. A: Chlorophyll *a* concentration. B: Phycocyanin concentration. C: *Microcystis* counts. Figure S2: Bar plots indicating microcystin concentration through time. (A) Total microcystins (B) Aqueous microcystins. Figure S3: Bar plots of the relative abundance of the top 20 more abundant bacterial families found during the study, grouped by hours after treatment.
