# Supplementary material for: Microbial Community Response to Granular Peroxide-Based Algaecide Treatment of a Cyanobacterial Harmful Algal Bloom in Lake Okeechobee, Florida (USA)
Source: Toxins (Basel). 2024 Apr 26;16(5):206. doi: 10.3390/toxins16050206 (PMC11125911; doi:10.3390/toxins16050206)
Supplement: Supplementary file 1 [file toxins-16-00206-s001.zip › toxins-2940842-supplementary.pdf]

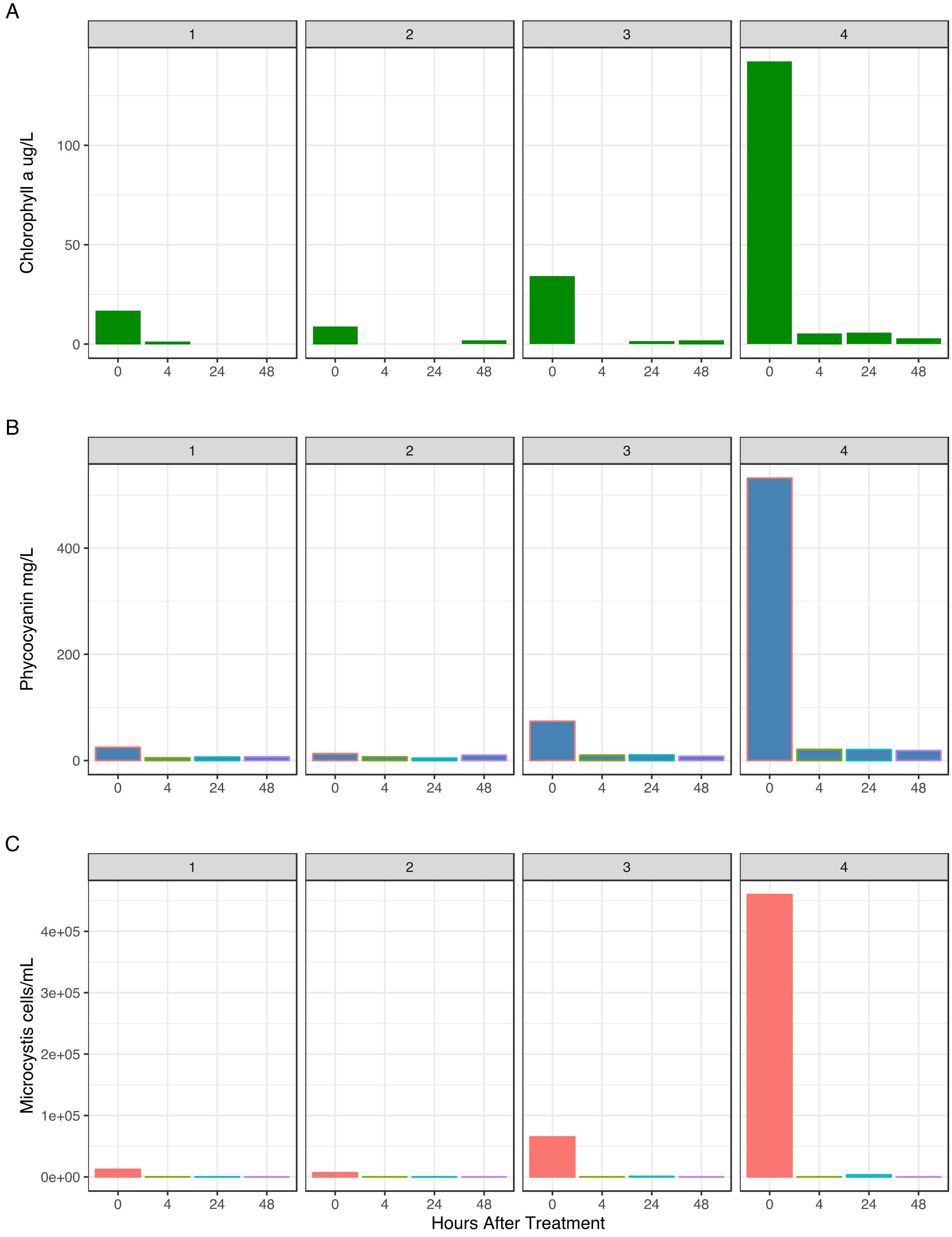

Figure S1: Bar plots indicating measures of cyanobacterial abundance grouped by location through time. A: Chlorophyll a concentration. B: Phycocyanin concentration. C: Microcystis counts.

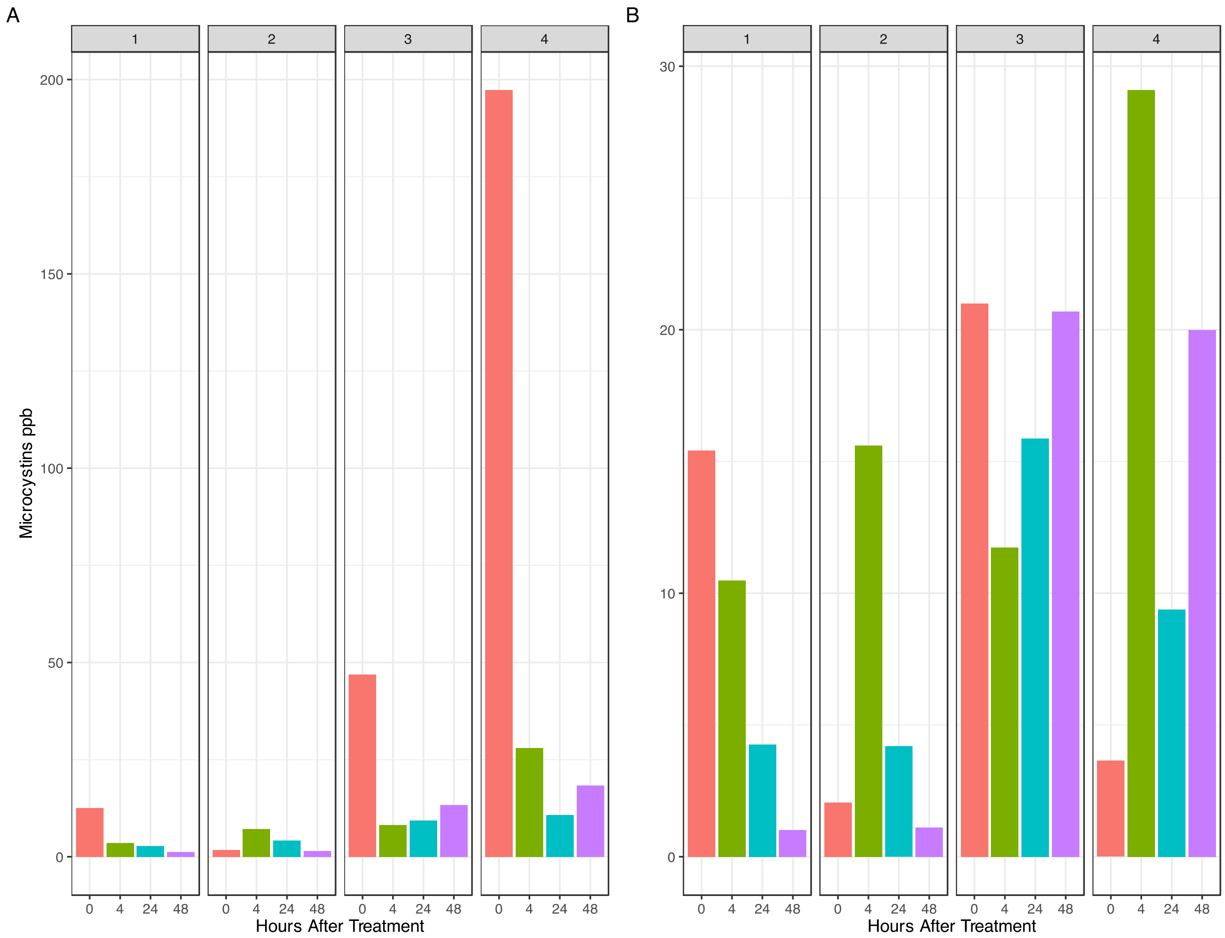

Figure S2: Bar plots indicating microcystin concentration through time. (A) Total microcystins (B) Aqueous microcystins.

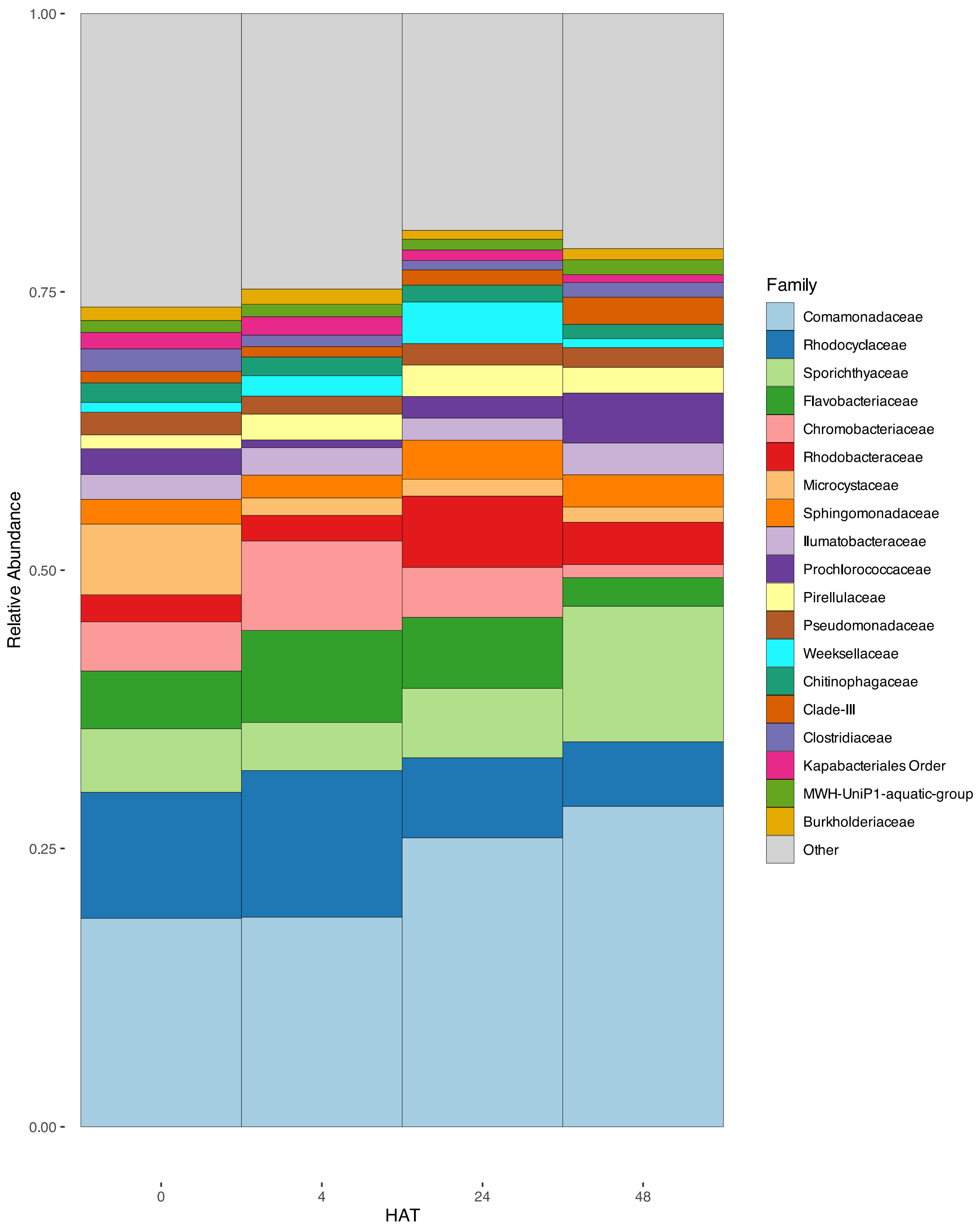

Figure S3: Bar plots of the relative abundance of the top 20 more abundant bacterial families found during the study, grouped by hours after treatment.
